# Camrelizumab-associated MG-like ocular neuromuscular toxicity after PD-1 inhibitor rechallenge in metastatic hepatocellular carcinoma: a case report with serial muscle injury-related biomarker changes

**DOI:** 10.3389/fimmu.2026.1908789

**Published:** 2026-08-05

**Authors:** Jie Li, Mengdie Chen, Lusheng Wang, Kesheng Bo, Ming Chen, Hui Jiang

**Affiliations:** 1 Gastroenterology Department, Wanbei Coal Electric Group General Hospital, Suzhou, Anhui, China; 2 Respiratory Department, Wanbei Coal Electric Group General Hospital, Suzhou, Anhui, China; 3 Central Laboratory, Wanbei Coal Electric Group General Hospital, Suzhou, Anhui, China

**Keywords:** Camrelizumab, immune-related adverse event, myasthenia gravis-like syndrome, muscle injury-related biomarkers, hepatocellular carcinoma

## Abstract

**Background:**

Immune checkpoint inhibitors (ICIs) can cause rare but clinically important immune-related neuromuscular adverse events. Early warning signals for ICI-associated neuromuscular toxicity remain poorly defined, particularly after PD-1 inhibitor rechallenge.

**Case presentation:**

We report a 71-year-old woman with metastatic hepatocellular carcinoma who developed progressive bilateral ptosis and blurred vision approximately 20 days after camrelizumab rechallenge following a prolonged treatment-free interval. Repetitive nerve stimulation of the left orbicularis oculi muscle showed a 24.8% decremental response, suggesting possible ocular neuromuscular junction involvement. Serial laboratory data showed progressive increases in muscle injury-related biomarkers, including CK, CK-MB, MYO, LDH, and α-HBDH, before hospitalization and before overt neurological deterioration. Cranial MRI and MRA excluded structural central nervous system lesions. Flow cytometric immune profiling showed descriptive changes in CD4+ T-cell parameters and granzyme B-positive CD8+ T-cell proportions, but these findings were not interpreted as evidence of causal immune activation. cTnI was measured using an immunoturbidimetric assay and did not exceed the laboratory-reported reference range; therefore, myocardial injury or ICI-associated myocarditis could not be established with the available data. Based on the temporal association with camrelizumab rechallenge, ocular manifestations, a suggestive RNS finding, serial muscle injury-related biomarker abnormalities, exclusion of structural central nervous system lesions, and clinical improvement after glucocorticoid therapy, camrelizumab-associated immune-related neuromuscular toxicity or an MG-like ocular syndrome was considered.

**Conclusions:**

This case highlights camrelizumab-associated MG-like ocular neuromuscular toxicity after PD-1 inhibitor rechallenge. Its main noteworthy feature is the availability of serial pre-hospitalization biomarker data showing progressive muscle injury-related biomarker abnormalities before overt neurological deterioration. Definite immune-related MG and ICI-associated myocarditis could not be established because confirmatory serological, electrophysiological, and cardiac investigations were incomplete. Longitudinal assessment of muscle injury-related biomarkers may help identify evolving immune-related neuromuscular toxicity in selected patients, although larger studies are needed for validation.

## 1 Introduction

Immune checkpoint inhibitors (ICIs) targeting the programmed cell death protein 1 (PD-1)/programmed death-ligand 1 (PD-L1) pathway have dramatically improved outcomes in a variety of malignancies, including hepatocellular carcinoma (HCC) (1–3). By restoring antitumor T-cell activity, PD-1 blockade can achieve durable clinical responses and prolonged survival. Camrelizumab, a humanized anti-PD-1 monoclonal antibody developed in China, has become an important therapeutic option for advanced HCC and several other solid tumors (4). However, enhanced immune activation induced by ICIs may simultaneously disrupt peripheral immune tolerance and trigger immune-related adverse events (irAEs), which can affect virtually any organ system (5).

Neurological irAEs are relatively uncommon, with an overall incidence generally below 5%, but they are among the most potentially devastating toxicities associated with ICIs because of their rapid progression and high mortality (6, 7). Reported neurological complications include peripheral neuropathy, encephalitis, aseptic meningitis, myositis, Guillain–Barré syndrome, and myasthenia gravis (MG)-like syndromes (8, 9). Among these entities, immune-related MG is particularly concerning because it frequently occurs together with skeletal muscle and cardiac involvement, forming a potentially fatal neuromuscular–cardiac overlap syndrome (10). Previous studies have reported mortality rates approaching 30–50% in patients with ICI-associated myocarditis–MG overlap syndromes, highlighting the importance of early recognition and intervention (11, 12).

Immune-related adverse events may also emerge or recur after ICI rechallenge, even following prolonged treatment-free intervals. Unlike conventional dose-dependent drug toxicity, irAEs are primarily mediated by immune dysregulation. Prior PD-1 blockade may induce durable immune remodeling, expansion of memory T-cell populations, and persistence of autoreactive or cross-reactive T-cell clones. After treatment discontinuation, peripheral immune tolerance may not be fully restored in susceptible individuals. Upon rechallenge, renewed PD-1 inhibition may rapidly amplify pre-existing autoreactive immune responses, promote epitope spreading, and trigger *de novo* or recurrent autoimmune tissue injury. These mechanisms provide a plausible immunological basis for clinically significant neuromuscular toxicity after re-exposure to PD-1 blockade, even after a prolonged treatment-free interval.

Unlike classical autoimmune MG, ICI-associated MG often exhibits atypical clinical features, rapid deterioration, and multisystem involvement (13). Early diagnosis of ICI-associated neuromuscular–cardiac overlap toxicity remains challenging because initial manifestations may be limited to nonspecific ocular symptoms, fatigue, or mild muscle discomfort, whereas life-threatening myocarditis, myositis, or respiratory failure may develop rapidly (14). Confirmatory tests, including neuromuscular autoantibodies, cardiac magnetic resonance imaging, and endomyocardial biopsy, are not always available in routine clinical practice, and electrophysiological studies are often performed only after neurological symptoms become evident. Moreover, validated early warning biomarkers for impending neuromuscular–cardiac toxicity have not been established. Although previous reports suggest that ICI-associated MG-like and neuromuscular overlap syndromes are frequently accompanied by muscle injury-related and cardiac evaluation biomarker abnormalities, the pre-symptomatic temporal evolution of these biomarkers remains poorly characterized in most published reports (15, 16).

Although neurological irAEs associated with nivolumab and pembrolizumab have been increasingly recognized, reports describing camrelizumab-associated neuromuscular toxicity remain relatively limited. Even fewer studies have documented toxicity occurring after immunotherapy rechallenge following a prolonged treatment interruption. In addition, data regarding the dynamic evolution of muscle injury-related biomarkers and immune profiling findings in such patients remain scarce.

Herein, we report a case of camrelizumab-associated immune-related neuromuscular toxicity or an MG-like ocular syndrome after PD-1 inhibitor rechallenge in a patient with metastatic HCC. Rather than representing a new clinical entity, this case is noteworthy for the availability of serial pre-hospitalization biomarker measurements showing progressive muscle injury-related biomarker abnormalities before overt neurological deterioration. This observation may help characterize the temporal evolution of biochemical abnormalities in selected patients receiving ICI rechallenge.

## 2 Case presentation

**Figure 1:**
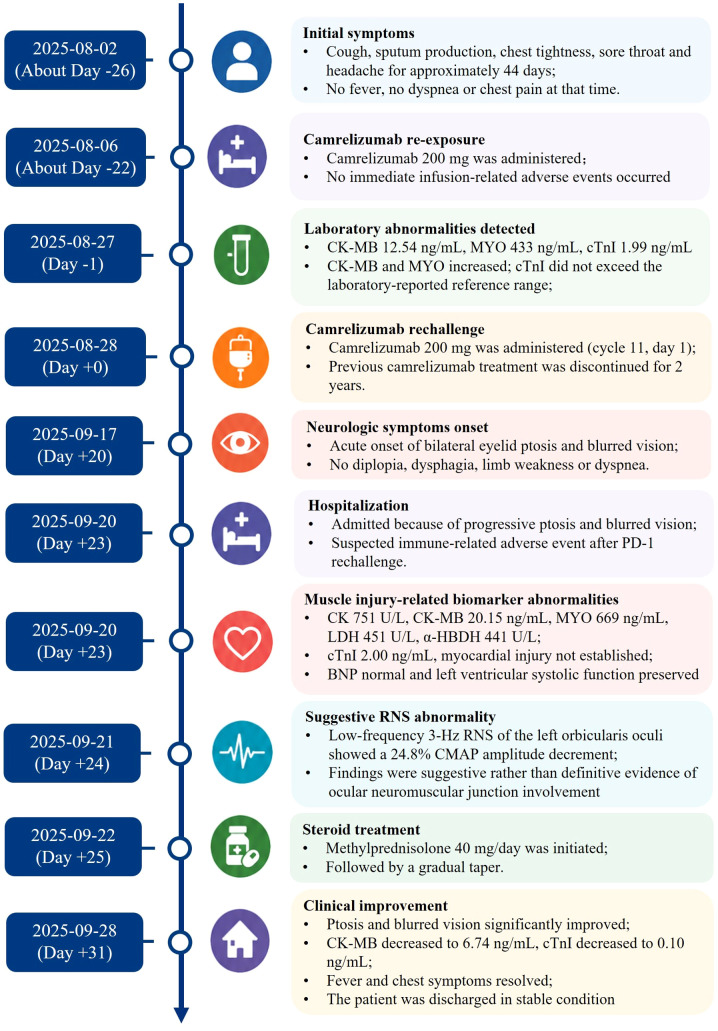
Clinical timeline of the patient. Timeline illustrating the clinical course from camrelizumab re-exposure and rechallenge to ocular symptom onset, diagnostic evaluation, glucocorticoid treatment, and clinical improvement.

A 71-year-old Chinese woman with a history of HCC was admitted to our department in September 2025 because of progressive bilateral ptosis and blurred vision after camrelizumab re-exposure for pulmonary metastatic disease. Before this index admission, she had been hospitalized from July 31 to August 11, 2025 for evaluation and management of pulmonary metastatic disease, during which camrelizumab was restarted on August 6, 2025. She was subsequently readmitted from August 27 to August 28, 2025 for short-term clinical evaluation and treatment planning. The overall clinical course is summarized in **Figure 1**.

The patient underwent right hepatectomy for primary HCC in March 2021. She subsequently received nine cycles of camrelizumab-based systemic therapy intermittently combined with tegafur and apatinib. Treatment was discontinued because of treatment-related intolerance, and she remained off systemic antitumor therapy for approximately two years. During the treatment-free interval, follow-up imaging and subsequent bronchoscopic biopsy confirmed pulmonary metastasis from HCC, providing the clinical basis for restarting camrelizumab-based therapy.

**Figure 2:**
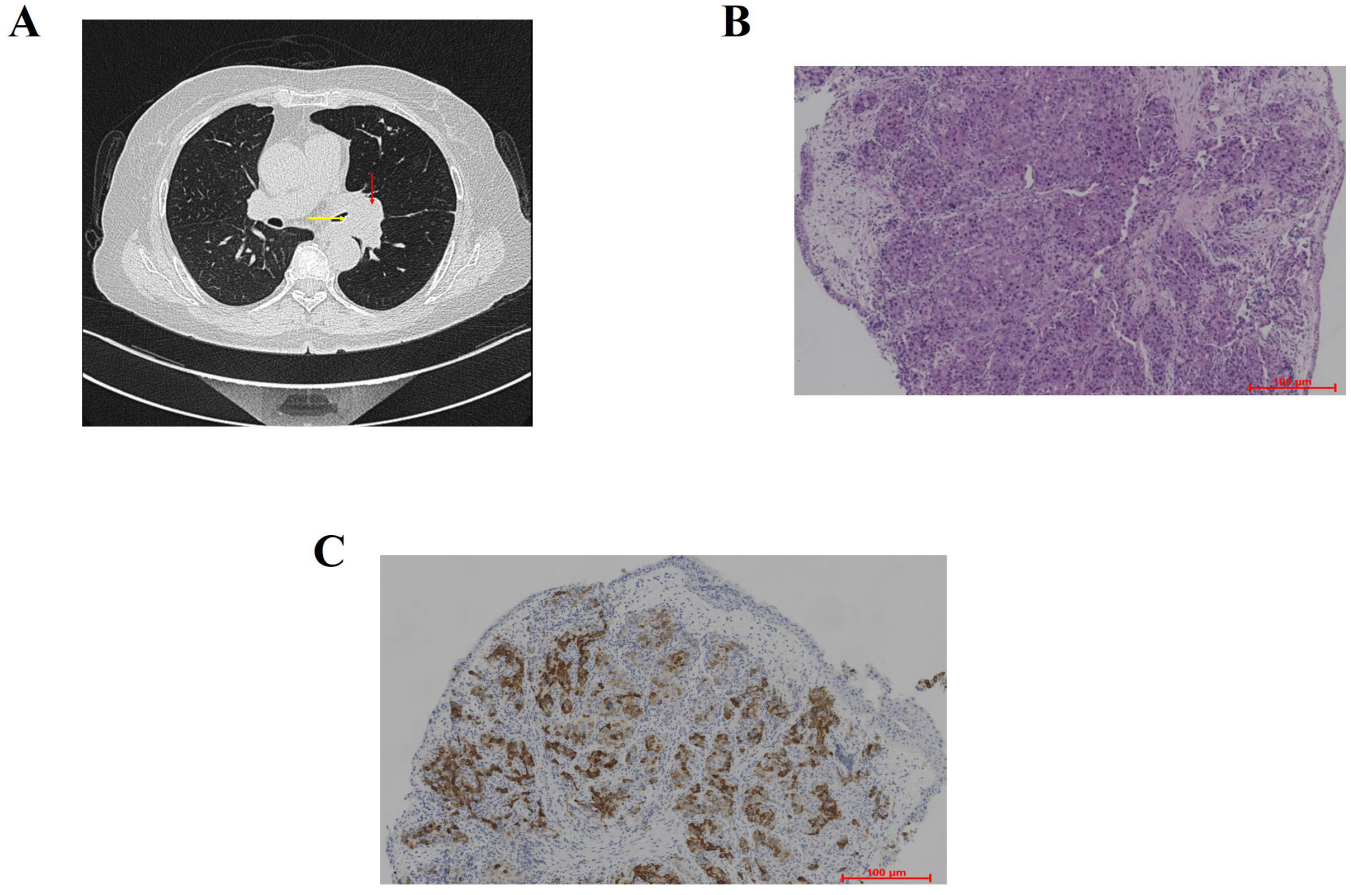
Radiological and pathological evidence of pulmonary metastatic HCC. **(A)** Chest computed tomography demonstrating a left hilar mass with narrowing and truncation of the left main bronchus; **(B)** Hematoxylin and eosin staining of the bronchoscopic biopsy specimen showing nests and trabecular arrangements of atypical tumor cells (original magnification ×10); **(C)** Positive HepPar-1 immunohistochemical staining supporting hepatocellular origin and confirming pulmonary metastasis from HCC.

Approximately 44 days before admission, the patient developed intermittent productive cough that was more pronounced at night and in the early morning, accompanied by throat discomfort, chest tightness, and headache. Chest computed tomography (CT) demonstrated enlargement of the left hilar shadow with truncation of the left main bronchus, multiple bilateral pulmonary nodules, chronic bronchitis, and emphysematous changes. Bronchoscopy revealed a lesion involving the left main bronchus. Histopathological examination confirmed malignant tumor infiltration. Immunohistochemical staining showed negativity for TTF-1, CK7, Napsin A, P63, P40, and CD56, whereas HepPar-1 was strongly positive and Arginase-1 demonstrated focal positivity. The Ki-67 proliferation index was approximately 20%, supporting the diagnosis of pulmonary metastasis from HCC (**Figure 2**).

After exclusion of treatment contraindications, camrelizumab was restarted during the hospitalization from July 31 to August 11, 2025, and the patient received camrelizumab 200 mg on August 6, 2025. No immediate infusion-related adverse events occurred. She was later readmitted from August 27 to August 28, 2025 for short-term clinical evaluation and received another dose of camrelizumab 200 mg on August 28, 2025. Approximately 20 days after the second camrelizumab administration, she gradually developed bilateral blurred vision, periorbital swelling, and progressive ptosis, which subsequently worsened and prompted the September 2025 admission.

On admission, physical examination revealed a body temperature of 36.2°C, pulse rate of 86 beats/min, respiratory rate of 23 breaths/min, and blood pressure of 123/82 mmHg. Bilateral ptosis, periorbital edema, and visual impairment were evident. Breath sounds over the left lung were decreased without obvious rales. Neurological examination revealed no limb weakness, sensory deficits, or pathological reflexes.

Laboratory investigations on the September admission showed elevations of several muscle injury-related biomarkers, including CK (751 U/L), CK-MB (20.15 ng/mL), LDH (451 U/L), α-HBDH (441 U/L), and MYO (669 ng/mL). cTnI was measured using an immunoturbidimetric assay, with a laboratory-reported reference range of 0.00–2.00 ng/mL. The measured cTnI value was 2.00 ng/mL and therefore reached but did not exceed the upper limit of the reported range. Because this was not a high-sensitivity troponin assay and no assay-specific 99th-percentile upper reference limit was available, low-level troponin release could not be reliably assessed. BNP remained within the normal range (**Table 1**).

**Table 1:** Key muscle injury-related biomarkers and available cardiac evaluation markers on admission.

Parameter	Result	Reference range	Unit
Creatine kinase (CK)	751 ↑	40–200	U/L
Creatine kinase-MB (CK-MB)	20.15 ↑	0–5.00	ng/mL
Lactate dehydrogenase (LDH)	451 ↑	120–250	U/L
α-Hydroxybutyrate dehydrogenase (α-HBDH)	441 ↑	72–182	U/L
Myoglobin (MYO)	669 ↑	0–60	ng/mL
Cardiac troponin-I (cTnI)	2.00	0.00–2.00	ng/mL
B-type natriuretic peptide (BNP)	6.2	0.0–100.0	pg/mL

The symbol ↑ indicates that the value is above the upper limit of the corresponding reference range.

**Figure 3:**
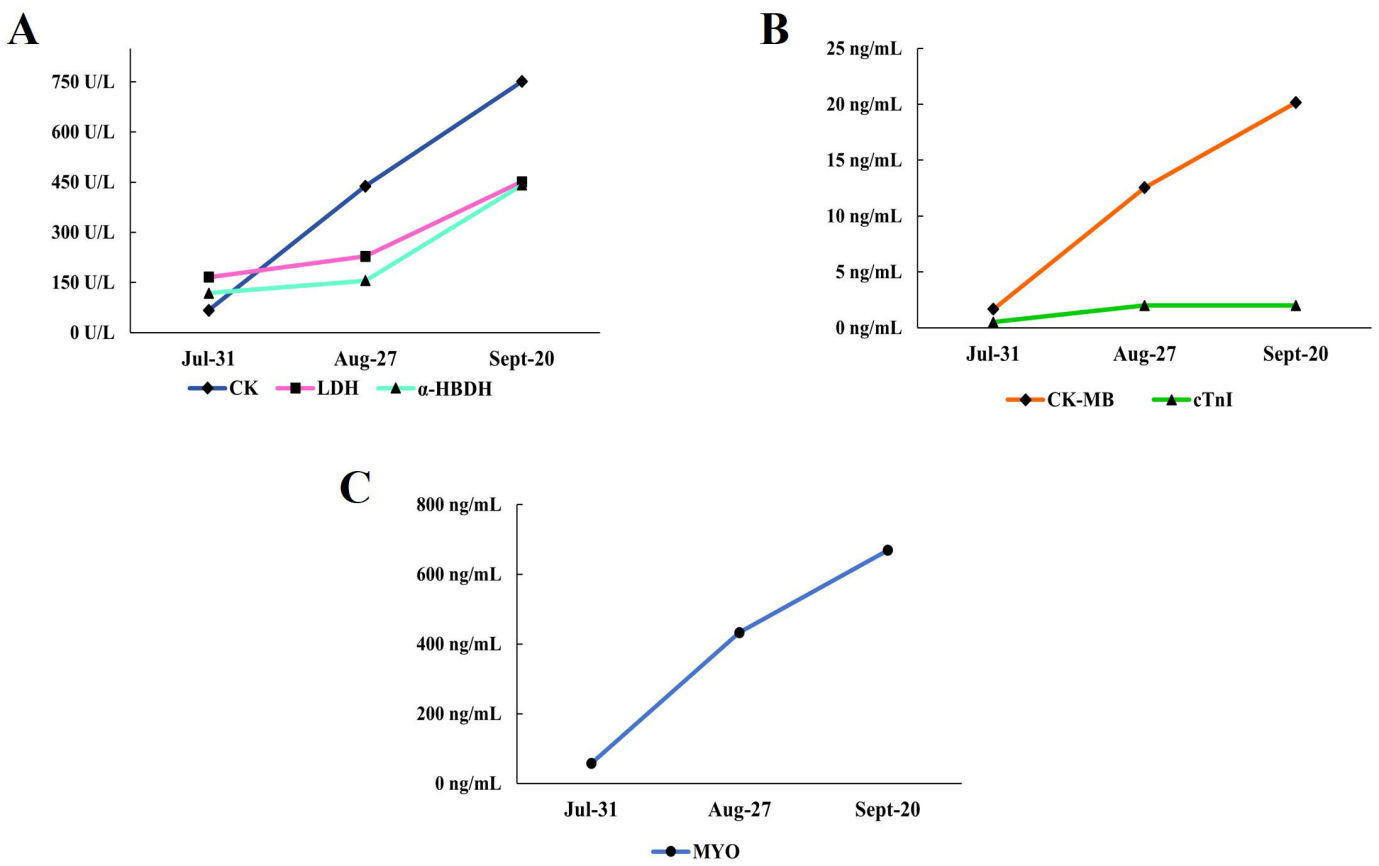
Dynamic changes in muscle injury-related biomarkers and available cTnI measurements before hospitalization. Serial laboratory measurements were obtained on July 31, August 27, and September 20, 2025. **(A)** Dynamic changes in CK, LDH, and α-HBDH. **(B)** Dynamic changes in CK-MB and cTnI. **(C)** Dynamic change in MYO. Serial measurements showed progressive increases in CK, CK-MB, MYO, LDH, and α-HBDH before hospitalization and before overt neurological deterioration. cTnI was measured using an immunoturbidimetric assay with a laboratory-reported reference range of 0.00–2.00 ng/mL and did not exceed this range.

Serial laboratory data were available from July 31, August 27, and September 20, 2025 (**Figure 3**). The July 31 measurements were obtained during the initial hospitalization as part of routine clinical assessment and pre-treatment evaluation before camrelizumab re-exposure. The August 27 results, obtained during short readmission before the September neurological presentation, already showed increased CK-MB (12.54 ng/mL) and MYO (433 ng/mL), with cTnI measuring 1.99 ng/mL using the same immunoturbidimetric assay. These findings indicated evolving pre-hospitalization muscle injury-related biomarker abnormalities before overt neurological deterioration, although myocardial injury could not be established from the available cTnI assay. Additional laboratory evaluation showed no clinically significant abnormalities in thyroid function, thyroid autoantibodies, coagulation parameters, or serum tumor markers (**Supplementary Tables 1**–**S4**).

Non-immune causes of muscle injury-related biomarker abnormalities were also considered. No statin exposure or other clearly myotoxic medication was documented in the available records during the index hospitalization, and prior apatinib and tegafur treatment had been discontinued approximately two years earlier. Infection-related or non-immune muscle injury was also considered because of respiratory symptoms and mild infectious changes on chest CT. Therefore, the enzyme abnormalities were interpreted as nonspecific muscle injury-related biomarker abnormalities rather than definitive evidence of immune-mediated myositis or myocarditis.

Flow cytometric immune profiling was available at three time points: July 31, August 27, and September 20, 2025 (**Table 2**). The August 27 panel showed a flow cytometry-derived total lymphocyte count of 2324 cells/µL, with increased CD4+ T-cell count (1859 cells/µL) and proportion (79.97%), as well as an increased proportion of granzyme B-positive CD8+ T cells (70.35%). On September 20, the CD4+ T-cell count and proportion were within the reference range, whereas the granzyme B-positive CD8+ T-cell proportion remained above the reference range. Because these measurements were descriptive and no functional, antigen-specific, or clonality assays were performed, the flow cytometry findings were not interpreted as direct evidence of causal immune activation.

**Table 2:** Longitudinal flow cytometric immune profiling at three clinical time points.

Parameter	31-Jul-25	27-Aug-25	20-Sep-25	Reference range
Total lymphocytes (/µL)	1835	2324	2574	1530–3700
CD4+ T cells (/µL)	992	1859 ↑	1145	455–1261
CD4+ T-cell proportion (%)	54.04 ↑	79.97 ↑	44.47	24.01–49.05
GzmB+ CD8+ T-cell proportion (%)	60.84	70.35 ↑	73.13 ↑	8.9–63.8

The symbol ↑ indicates that the value is above the upper limit of the corresponding reference range.

Electrocardiography demonstrated sinus rhythm with low QRS voltage in the limb leads. Transthoracic echocardiography revealed preserved left ventricular systolic function, impaired left ventricular diastolic function, mild aortic valve calcification with trivial regurgitation, and moderate tricuspid regurgitation. No overt heart failure was identified, and B-type natriuretic peptide levels remained within the normal range.

Given the possibility of irAEs, multidisciplinary consultations involving cardiology, neurology, and ophthalmology were undertaken. Cardiology consultation was requested because of elevated CK-MB and MYO, low QRS voltage, and concern for possible myocardial injury in the clinical record. However, myocardial injury or myocarditis could not be established because cTnI did not exceed the laboratory-reported reference range, the available assay could not reliably assess low-level troponin release, BNP was normal, left ventricular systolic function was preserved, and cardiac MRI or endomyocardial biopsy was not performed.

**Figure 4:**
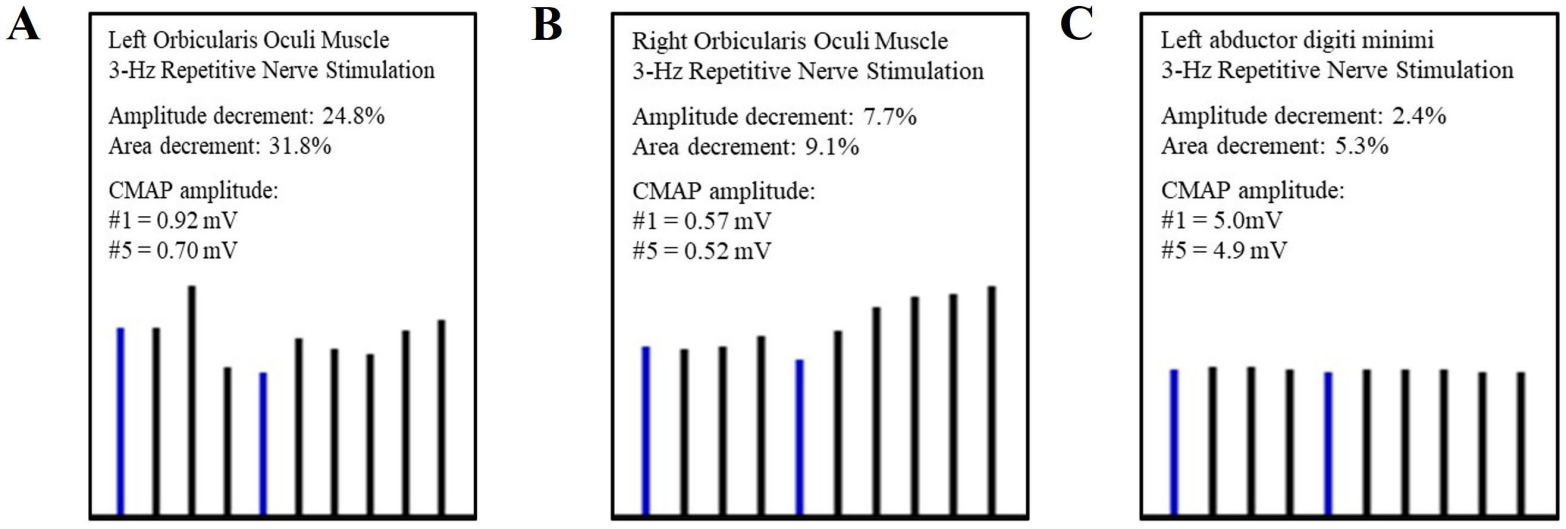
Repetitive nerve stimulation findings. Representative 3-Hz RNS studies were performed in the bilateral orbicularis oculi muscles and the left abductor digiti minimi muscle. **(A)** RNS of the left orbicularis oculi muscle demonstrated a significant decremental response, with a 24.8% reduction in CMAP amplitude and a 31.8% reduction in CMAP area between the first and fifth responses, suggesting possible ocular neuromuscular junction involvement; **(B)** RNS of the right orbicularis oculi muscle showed no significant decremental response, with a 7.7% CMAP amplitude decrement and a 9.1% CMAP area decrement; **(C)** RNS of the left abductor digiti minimi muscle showed no significant decremental response, with a 2.4% CMAP amplitude decrement and a 5.3% CMAP area decrement. A CMAP amplitude decrement greater than 10% was considered abnormal. Blue bars indicate the first and fifth CMAP responses.

Electrophysiological studies demonstrated normal bilateral facial nerve compound muscle action potentials and normal blink reflexes. The revised analysis focused on the 3-Hz low-frequency RNS results, which are the standard frequency range used to assess a myasthenic decrement. Low-frequency RNS at 3 Hz was performed in the left orbicularis oculi, right orbicularis oculi, and left abductor digiti minimi muscles. A significant decremental response was observed only in the left orbicularis oculi muscle, with a 24.8% reduction in compound muscle action potential amplitude, exceeding the conventional abnormal threshold of 10%. In contrast, the right orbicularis oculi muscle showed a 7.7% decrement and the left abductor digiti minimi muscle showed a 2.4% decrement, both below the abnormal threshold (**Figure 4**).

Preserved pupillary light reflexes, unremarkable thyroid testing, normal cranial MRI/MRA, normal facial nerve CMAPs, and normal blink reflexes made structural central nervous system disease, thyroid-related disease, and facial neuropathy less likely. However, because detailed ocular motility and fatigability testing were not documented, alternative causes such as pupil-sparing microvascular ocular motor palsy and age-related aponeurotic ptosis could not be definitively excluded. Therefore, the ocular syndrome was interpreted mainly on the basis of the clinical presentation, temporal association with camrelizumab exposure, and a single asymmetric low-frequency RNS abnormality, and the diagnosis was regarded as an MG-like ocular syndrome rather than definite immune-related MG.

Based on the temporal association between camrelizumab exposure and ocular symptom progression, characteristic ocular manifestations, a suggestive unilateral low-frequency RNS abnormality, exclusion of structural central nervous system lesions, dynamic muscle injury-related biomarker abnormalities, and clinical improvement after glucocorticoid therapy, camrelizumab-associated immune-related neuromuscular toxicity or an MG-like ocular syndrome was considered rather than definite immune-related MG. Myocardial injury or ICI-associated myocarditis could not be established with the available data.

The patient received intravenous methylprednisolone at 40 mg daily during hospitalization, together with myocardial protective therapy, neurotrophic agents, anti-infective treatment, expectorants, and supportive care. During treatment, cough and sputum production improved, and ptosis and blurred vision partially resolved. Repeat laboratory testing showed substantial decreases in CK-MB, MYO, LDH, and α-HBDH levels. After eight days of hospitalization, the patient was discharged in stable condition on September 28, 2025, with oral prednisone, coenzyme Q10, methylcobalamin, and close outpatient follow-up.

## 3 Discussion

ICIs have transformed the therapeutic landscape of advanced malignancies, including HCC, by restoring antitumor T-cell immunity (17, 18). However, disruption of immune tolerance may also lead to irAEs, which can affect virtually any organ system. Although neurological irAEs occur in fewer than 5% of patients receiving ICIs, they are associated with disproportionately high morbidity and mortality, particularly when accompanied by cardiac or skeletal muscle involvement (19, 20).

In the present case, a patient with metastatic HCC developed progressive bilateral ptosis and blurred vision approximately three weeks after camrelizumab rechallenge. RNS demonstrated a significant decremental response in the left orbicularis oculi muscle, whereas cranial MRI and MRA were unremarkable. Concurrently, marked elevations of CK, CK-MB, MYO, LDH, and α-HBDH were observed. Importantly, both neurological symptoms and biochemical abnormalities improved following glucocorticoid therapy. Collectively, these findings suggested camrelizumab-associated immune-related neuromuscular toxicity or an MG-like ocular syndrome. Myocardial injury or ICI-associated myocarditis could not be established with the available data.

One of the major diagnostic challenges in this case was differentiating immune-related neuromuscular toxicity from alternative causes of ptosis and visual impairment. Central nervous system metastasis was excluded by contrast-enhanced MRI and MRA. Facial nerve conduction studies and blink reflex examinations were normal, arguing against peripheral cranial neuropathy. In contrast, the positive RNS finding localized the pathological process to the neuromuscular junction. Although AChR, MuSK, and anti-striational antibody testing and single-fiber EMG were unavailable, the ocular manifestations, temporal association with camrelizumab exposure, unilateral low-frequency RNS abnormality, exclusion of structural central nervous system lesions, and favorable steroid responsiveness supported an MG-like ocular syndrome, but did not establish definite immune-related MG.

Neurological irAEs encompass a broad spectrum of disorders, including peripheral neuropathy, encephalitis, aseptic meningitis, myositis, and immune-related MG (21). ICI-associated MG is clinically important because it may occur as part of an overlap syndrome involving myositis and myocarditis. Previous studies have shown that ICI-associated MG can differ from idiopathic MG, with more rapid progression, early respiratory compromise, and frequent coexistence of skeletal muscle or cardiac injury (13). In severe and confirmed myocarditis–MG overlap syndromes, high mortality rates have been reported, emphasizing the importance of early recognition and intervention. However, the present patient did not have confirmed myocarditis, overt heart failure, respiratory failure, or a fulminant clinical course. Therefore, the high mortality reported for severe overlap syndromes should be interpreted as general background information rather than as directly applicable to this case.

The cardiac findings in this case should be interpreted with particular caution. CK-MB and MYO can increase in skeletal muscle injury, whereas LDH and α-HBDH are nonspecific markers of tissue injury. The available cTnI was measured using an immunoturbidimetric assay with a laboratory-reported reference range of 0.00–2.00 ng/mL. The measured value reached but did not exceed this range, and no assay-specific 99th-percentile upper reference limit was available. Therefore, this result could not establish myocardial injury and could not reliably exclude low-level troponin release. In addition, BNP remained normal, left ventricular systolic function was preserved, and neither cardiac MRI nor endomyocardial biopsy was performed. Accordingly, myocardial injury or ICI-associated myocarditis could not be established with the available data.

To better contextualize the present case, we summarized previously published cases and relevant case series of ICI-associated neurological irAEs and neuromuscular–cardiac overlap toxicities in **Supplementary Table 6**. A noteworthy aspect of this case was the availability of serial pre-hospitalization biomarker data before overt neurological deterioration. Unlike many reported cases in which biomarker abnormalities are first identified at clinical presentation, our patient showed progressive increases in CK, CK-MB, MYO, LDH, and α-HBDH before hospitalization and before the full development of clinically apparent neuromuscular dysfunction. This trajectory suggests that muscle injury-related biomarker abnormalities may precede clinically apparent neuromuscular deterioration in selected patients, but this observation remains hypothesis-generating.

From a translational perspective, this case suggests that longitudinal assessment of CK, CK-MB, MYO, LDH, α-HBDH, and cTnI may help identify evolving muscle injury-related abnormalities in selected patients receiving PD-1 inhibitor rechallenge. However, this observation is based on a single retrospective case and should not be interpreted as evidence supporting routine surveillance for all patients. Unexplained biomarker elevation or rapid upward trends, particularly when accompanied by ocular or muscle symptoms, should prompt timely neurological and cardiological assessment. Larger prospective studies are needed to define appropriate monitoring intervals, warning thresholds, and prognostic value.

Longitudinal flow cytometric immune profiling showed descriptive changes in CD4+ T-cell parameters and granzyme B-positive CD8+ T-cell proportions across three available time points. The August 27 panel showed increased CD4+ T-cell count and proportion, whereas the September 20 panel showed normalization of CD4+ T-cell parameters but persistent elevation of the granzyme B-positive CD8+ T-cell proportion. Because no functional, antigen-specific, or clonality assays were performed, these findings should be interpreted descriptively and should not be regarded as direct evidence of causal immune activation.

The immune context of HCC may also be influenced by its underlying etiology. HBV- and HCV-related HCC arise in the setting of chronic viral antigen exposure, persistent hepatic inflammation, altered immune tolerance, and virus-specific T-cell exhaustion, whereas NASH-related HCC is characterized by metabolic inflammation, innate immune activation, and distinct patterns of T-cell dysfunction (22, 23). These etiological differences may affect both antitumor immune responses and susceptibility to immune-mediated adverse events during PD-1 blockade. Although the present case does not allow definitive etiological stratification of irAE risk, future studies should evaluate whether HBV-, HCV-, and NASH-related HCC differ in their susceptibility to neuromuscular–cardiac irAEs after ICI therapy or rechallenge.

Camrelizumab is widely used in China for the treatment of HCC and several other malignancies (24). While dermatologic and endocrine irAEs are well recognized, reports describing camrelizumab-associated neuromuscular toxicity remain relatively uncommon. Furthermore, most published cases focus on either isolated MG-like syndrome or isolated myocarditis. The present case adds to the clinical observations of camrelizumab-associated neuromuscular toxicity by describing an ocular-predominant MG-like syndrome accompanied by dynamic muscle injury-related biomarker abnormalities. Importantly, toxicity developed after re-exposure to camrelizumab following a prolonged treatment interruption, highlighting that clinically significant irAEs may occur even in patients who previously tolerated PD-1 blockade.

The occurrence of severe toxicity after a prolonged treatment-free interval may be explained by durable immune remodeling induced by prior PD-1 blockade. Persistent memory T-cell populations or autoreactive T-cell clones may remain clinically silent after treatment discontinuation but can be rapidly reactivated upon PD-1 inhibitor rechallenge. Renewed checkpoint inhibition may lower the threshold for autoreactive T-cell activation, promote epitope spreading, and amplify tissue-directed cytotoxic immune responses, thereby contributing to *de novo* or recurrent autoimmune injury. In addition, emerging evidence suggests that gut microbiota composition may influence both antitumor immunity and susceptibility to irAEs by shaping systemic T-cell activation, cytokine production, and mucosal immune tolerance (25). Although microbiome profiling was not performed in our patient, these mechanisms provide a plausible biological framework for understanding severe immune-mediated toxicity after camrelizumab rechallenge, even after a prolonged treatment-free interval.

This case has several limitations. First, AChR, MuSK, and anti-striational antibodies were not assessed, and single-fiber electromyography was not performed, preventing definitive confirmation of immune-related MG. Second, myocardial injury or ICI-associated myocarditis could not be established. CK-MB and MYO are not specific for myocardium, and LDH and α-HBDH are nonspecific markers of tissue injury. cTnI was measured using an immunoturbidimetric assay with a laboratory-reported reference range of 0.00–2.00 ng/mL, and the measured value reached but did not exceed this range. Because this was not a high-sensitivity troponin assay and no assay-specific 99th-percentile upper reference limit was available, low-level troponin release could not be reliably assessed. In addition, BNP remained normal, left ventricular systolic function was preserved, and neither cardiac MRI nor endomyocardial biopsy was performed. Third, this was a single retrospective case, and serial immunophenotyping, complete ocular fatigability assessment, and long-term follow-up data were unavailable, limiting further mechanistic and prognostic interpretation.

Nevertheless, this case underscores several important clinical messages. New-onset ptosis, diplopia, or visual symptoms in patients receiving ICIs should prompt immediate evaluation for immune-related neuromuscular toxicity. Concurrent assessment of CK, CK-MB, MYO, LDH, α-HBDH, and cTnI may help identify evolving muscle injury-related abnormalities and prompt timely neurological and cardiological assessment, but these biomarkers alone should not be considered diagnostic of myocardial involvement. RNS remains a valuable diagnostic tool when antibody testing is unavailable, and multidisciplinary collaboration among oncologists, neurologists, cardiologists, and ophthalmologists is essential for timely diagnosis and treatment. Early recognition and prompt immunosuppressive therapy may substantially improve outcomes in patients with severe neurological irAEs.

## 4 Conclusion

We report a case of camrelizumab-associated immune-related neuromuscular toxicity or an MG-like ocular syndrome after PD-1 inhibitor rechallenge in metastatic HCC. The main noteworthy feature was the availability of serial pre-hospitalization biomarker data showing progressive increases in CK, CK-MB, MYO, LDH, and α-HBDH before overt neurological deterioration. Definite immune-related MG could not be established because serological testing and single-fiber electromyography were unavailable. Myocardial injury or ICI-associated myocarditis could also not be established because the available cTnI assay was an immunoturbidimetric assay that did not exceed the laboratory-reported reference range, BNP was normal, left ventricular systolic function was preserved, and cardiac MRI or endomyocardial biopsy was not performed. This case suggests that serial muscle injury-related biomarker monitoring and timely neurological assessment may help identify evolving immune-related neuromuscular toxicity in selected patients receiving PD-1 inhibitor rechallenge.

## Data Availability

The original contributions presented in the study are included in the article/**Supplementary Material**. Further inquiries can be directed to the corresponding author.
