# Supplementary material for: Camrelizumab-associated MG-like ocular neuromuscular toxicity after PD-1 inhibitor rechallenge in metastatic hepatocellular carcinoma: a case report with serial muscle injury-related biomarker changes
Source: Front Immunol. 2026 Aug 5;17:1908789. doi: 10.3389/fimmu.2026.1908789 (PMC13485550; doi:10.3389/fimmu.2026.1908789)
Supplement: Supplementary file 1 [file DataSheet1.docx]

**Supporting Information**

**Camrelizumab-Associated MG-like Ocular Neuromuscular Toxicity After PD-1 Inhibitor Rechallenge in Metastatic Hepatocellular Carcinoma: A Case Report with Serial Pre-Hospitalization Biomarker Changes**

Jie Li^1^, Mengdie Chen^2^, Lusheng Wang^3^, Kesheng Bo^2^, Ming Chen^2†^, Hui Jiang^2†^

^1^Gastroenterology Department, Wanbei Coal Electric Group General Hospital, Suzhou, Anhui 234000, China, ^2^Respiratory Department, Wanbei Coal Electric Group General Hospital, Suzhou, Anhui Province 234000, China, ^3^Central Laboratory, Wanbei Coal Electric Group General Hospital, Suzhou, Anhui Province 234000, China

Table **S1**. Selected hematological findings on admission

| **Parameter** | **Result** | **Reference Range** | **Interpretation** |
| --- | --- | --- | --- |
| White blood cell count (WBC) | 4.69 | 3.50–9.50 ×10⁹/L | Normal |
| Absolute lymphocyte count (LYMPH#) | 1.07 ↓ | 1.10–3.20 ×10⁹/L | Mild lymphopenia |
| Hemoglobin (HGB) | 142 | 115–150 g/L | Normal |
| Platelet count (PLT) | 227 | 125–350 ×10⁹/L | Normal |

Table **S2**. Thyroid function and thyroid autoantibody assessment on admission

| **Parameter** | **Result** | **Reference Range** | **Interpretation** |
| --- | --- | --- | --- |
| Free triiodothyronine (FT3) | 5.04 | 2.77–6.31 pmol/L | Normal |
| Free thyroxine (FT4) | 16.1 | 10.44–24.38 pmol/L | Normal |
| Thyroid-stimulating hormone (TSH) | 2.7 | 0.38–4.34 mIU/L | Normal |
| Thyroid peroxidase antibody (TPO-Ab) | 41.1 | 0.0–60.0 IU/mL | Negative |
| Anti-thyroglobulin antibody (ATG-Ab) | <1.3 | 0.0–4.5 IU/mL | Negative |

Table **S3**. Coagulation parameters on admission

| **Parameter** | **Result** | **Reference Range** | **Interpretation** |
| --- | --- | --- | --- |
| Activated partial thromboplastin time (APTT) | 22.2 ↓ sec | 22.3–32.5 sec | Mild shortening |
| Fibrinogen (FIB) | 3.54 g/L | 2.00–4.00 g/L | Normal |
| D-dimer | 0.58 ↑ mg/L (FEU) | 0.00–0.55 mg/L (FEU) | Mildly elevated |
| Fibrin degradation products (FDP) | 2.22 mg/L | 0.00–5.00 mg/L | Normal |

Table **S4**. Tumor marker profile on admission

| **Biomarker** | **Result** | **Reference Range** | **Interpretation** |
| --- | --- | --- | --- |
| Neuron-specific enolase (NSE) | 23.3 ↑ ng/mL | 0.0–15.7 ng/mL | Mildly elevated |
| Squamous cell carcinoma antigen (SCCA) | 0.7 ng/mL | 0.0–2.5 ng/mL | Normal |
| Cytokeratin 19 fragment (CYFRA21-1) | 2.1 ng/mL | 0.0–7.0 ng/mL | Normal |
| Carcinoembryonic antigen (CEA) | 1.7 ng/mL | 0.0–5.0 ng/mL | Normal |

Table **S5**. Comprehensive flow cytometric immune cell profiling results

| **Parameter** | **Result** | **Reference Range** | **Unit** | **Interpretation** |
| --- | --- | --- | --- | --- |
| Lymphocytes (CD45+) | 2574 | 1530–3700 | cells/μL | Normal |
| T cells (CD45+CD3+) | 1572 | 876–2310 | cells/μL | Normal |
| NK cells (CD45+CD3−CD16+CD56+) | 358 | 109–780 | cells/μL | Normal |
| Cytotoxic T lymphocytes (CTL, CD8+) | 427 | 258–958 | cells/μL | Normal |
| Helper T cells (Th, CD4+) | 1145 | 455–1261 | cells/μL | High-normal |
| T cells (%) | 61.07 | 53.33–81.22 | % | Normal |
| NK cells (%) | 13.89 | 6.40–34.83 | % | Normal |
| CTL (%) | 16.6 | 15.71–38.24 | % | Low-normal |
| Helper T cells (%) | 44.47 | 24.01–49.05 | % | Elevated tendency |
| Granzyme B+ NK cells | 274 | 74–526 | cells/μL | Normal |
| IFN-γ+ NK cells | 61 | 34–245 | cells/μL | Normal |
| Granzyme B+ CD8+ T cells | 73 ↓ | 91–445 | cells/μL | Reduced absolute count |
| IFN-γ+ CD8+ T cells | 312 | 71–541 | cells/μL | Normal |
| Granzyme B+ NK cells (%) | 76.66 | 45.4–94.5 | % | Normal |
| IFN-γ+ NK cells (%) | 32.02 | 14.6–55.1 | % | Normal |
| Granzyme B+ CD8+ T cells (%) | 73.13 ↑ | 8.9–63.8 | % | Enhanced cytotoxic activity |
| IFN-γ+ CD8+ T cells (%) | 17.18 | 11.7–52.4 | % | Normal |

Table **S6**.Published cases and series of ICI-associated neurological irAEs and neuromuscular–cardiac overlap toxicities

| **First author/year** | **Tumor type** | **ICI regimen/ rechallenge** | **Neurological phenotype** | **Muscle/cardiac involvement** | **Diagnostic evidence** | **Biomarker timing/trajectory** | **Treatment and outcome** |
| --- | --- | --- | --- | --- | --- | --- | --- |
| Published case 1 **^[1, 2]^** | NR | Camrelizumab-based therapy; rechallenge NR | MG-like syndrome / myositis / neuropathy / encephalitis / GBS-like syndrome | Present / absent / NR | RNS/EMG and antibody data: positive / negative / NR | Biomarkers were reported after neurological symptoms or were not serially monitored; no evidence of pre-symptomatic elevation | Corticosteroids ± IVIG / plasma exchange; improved / fatal / NR |
| Published case 2 **^[3]^** | NR | Camrelizumab alone or in combination; no rechallenge / NR | Neurological irAE | Present / absent / NR | RNS/EMG or antibody data: NR | CK, CK-MB, MYO, cTnI, LDH, or α-HBDH were reported at presentation; serial pre-symptomatic monitoring was not available | Corticosteroids ± supportive therapy; improved / poor outcome / NR |
| Published case 3 **^[4, 5]^** | NR | Camrelizumab plus chemotherapy or targeted therapy; no rechallenge / NR | Neuromuscular or central nervous system toxicity | Present / absent / NR | RNS/EMG and antibody data: positive / negative / NR | Dynamic biomarker changes were not reported or were recorded only after symptom onset | Drug discontinuation, corticosteroids, IVIG, or supportive care; outcome NR |
| Published case 4 **^[6-8]^** | NR | Other PD-1/PD-L1 inhibitor; rechallenge NR | MG / myositis / myocarditis overlap | Myocardial and/or skeletal muscle involvement present | EMG/RNS, cardiac evaluation, or antibody testing reported / NR | Biomarker elevation was usually detected after clinical manifestations; no clear pre-symptomatic trajectory was documented | Corticosteroids ± IVIG / plasma exchange / immunosuppression; variable outcome |
| Present case | Metastatic HCC with pulmonary metastasis | Camrelizumab rechallenge after prolonged treatment-free interval; previous camrelizumab-based therapy intermittently combined with apatinib and tegafur | Progressive bilateral ptosis and blurred vision approximately 20 days after rechallenge; MG-like ocular neuromuscular junction dysfunction | Clinically suspected myocardial involvement and skeletal muscle injury | RNS showed 24.5% CMAP amplitude decrement in the left orbicularis oculi; AChR/MuSK not tested | Progressive CK, CK-MB, MYO, cTnI, LDH, and α-HBDH elevation before hospitalization and before overt neurological deterioration | Intravenous methylprednisolone and supportive therapy; partial symptom improvement and biomarker decrease |

1.	Safa H, Johnson DH, Trinh VA, Rodgers TE, Lin H, Suarez-Almazor ME, Fa'ak F, Saberian C, Yee C, Davies MA *et al*: Immune checkpoint inhibitor related myasthenia gravis: single center experience and systematic review of the literature. *Journal for immunotherapy of cancer* 2019, 7(1):319.

2.	Makarious D, Horwood K, Coward JIG: Myasthenia gravis: An emerging toxicity of immune checkpoint inhibitors. *Eur J Cancer* 2017, 82:128-136.

3.	Suzuki S, Ishikawa N, Konoeda F, Seki N, Fukushima S, Takahashi K, Uhara H, Hasegawa Y, Inomata S, Otani Y *et al*: Nivolumab-related myasthenia gravis with myositis and myocarditis in Japan. *Neurology* 2017, 89(11):1127-1134.

4.	Kao JC, Liao B, Markovic SN, Klein CJ, Naddaf E, Staff NP, Liewluck T, Hammack JE, Sandroni P, Finnes H *et al*: Neurological Complications Associated With Anti-Programmed Death 1 (PD-1) Antibodies. *JAMA Neurol* 2017, 74(10):1216-1222.

5.	Johansen A, Christensen SJ, Scheie D, Højgaard JLS, Kondziella D: Neuromuscular adverse events associated with anti-PD-1 monoclonal antibodies: Systematic review. *Neurology* 2019, 92(14):663-674.

6.	Moreira A, Loquai C, Pföhler C, Kähler KC, Knauss S, Heppt MV, Gutzmer R, Dimitriou F, Meier F, Mitzel-Rink H *et al*: Myositis and neuromuscular side-effects induced by immune checkpoint inhibitors. *Eur J Cancer* 2019, 106:12-23.

7.	Pathak R, Katel A, Massarelli E, Villaflor VM, Sun V, Salgia R: Immune Checkpoint Inhibitor-Induced Myocarditis with Myositis/Myasthenia Gravis Overlap Syndrome: A Systematic Review of Cases. *Oncologist* 2021, 26(12):1052-1061.

8.	Wai Siu DH, O'Neill RS, Harris CA, Wang J, Ardolino L, Downton T, Tong M, Hong JH, Chin V, Clingan PR *et al*: Immune checkpoint inhibitor-induced myocarditis, myositis, myasthenia gravis and transaminitis: a case series and review. *Immunotherapy* 2022, 14(7):511-520.
